# Transcriptome analysis and identification of abscisic acid and gibberellin-related genes during seed development of alfalfa (*Medicago sativa* L.)

**DOI:** 10.1186/s12864-022-08875-0

**Published:** 2022-09-13

**Authors:** Lu Zhao, Mingyu Li, Xiaomei Ma, Dong Luo, Qiang Zhou, Wenxian Liu, Zhipeng Liu

**Affiliations:** The State Key Laboratory of Grassland Agro-ecosystems, Key Laboratory of Grassland Livestock Industry Innovation, Ministry of Agriculture and Rural Affairs, Engineering Research Center of Grassland Industry, Ministry of Education, College of Pastoral Agriculture Science and Technology, Lanzhou University, Lanzhou, 730020 People’s Republic of China

**Keywords:** Alfalfa, Seed development, Transcriptome analysis, Hormone, Abscisic acid, Gibberellin

## Abstract

**Background:**

Alfalfa (*Medicago sativa*) is a widely cultivated plant. Unlike many crops, the main goal of breeding alfalfa is to increase its aboveground biomass rather than the biomass of its seeds. However, the low yield of alfalfa seeds limits alfalfa production. Many studies have explored the factors affecting seed development, in which phytohormones, especially ABA and GAs, play an important role in seed development.

**Results:**

Here, we performed a transcriptome analysis of alfalfa seeds at five development stages. A total of 16,899 differentially expressed genes (DEGs) were identified and classified into 10 clusters, and the enriched Gene Ontology (GO) terms and Kyoto Encyclopedia of Genes and Genomes (KEGG) pathways were determined. The contents of ABA, GA_1_, GA_3_, GA_4_ and GA_7_ in alfalfa seeds at five development stages were determined. In addition, 14 ABA-related DEGs and 20 GA-related DEGs were identified and analysed. These DEGs are involved in plant hormone pathways and play an important role in seed development. Moreover, morphological and physiological analyses revealed the dynamic changes during the development of alfalfa seeds.

**Conclusion:**

Overall, our study is the first to analyse the transcriptome across various stages of seed development in alfalfa. The results of our study could be used to improve alfalfa seed yield. The key ABA and GA related-genes are potential targets for improving alfalfa seed yield via genetic engineering in the future.

**Supplementary Information:**

The online version contains supplementary material available at 10.1186/s12864-022-08875-0.

## Background

The seed is not only a reproductive organ of plants but also the source of nourishment of the embryo giving rise to a new plant [1]. The seed development process can be divided into two stages. The first is the differentiation and development of tissues and organs, and the second is the accumulation of nutrients and seed maturation. Both of these processes are highly complex [2]. After the seed becomes fully mature, the dry matter content remains stable, the water content decreases, and the embryo enters a dormant state [3]. During seed development, large amounts of nutrients are synthesized and stored, including various carbohydrates, proteins, and fats [4]. The development of the seed not only affects seed quality but also affects the normal growth and development of the following generation. Many studies have evaluated the factors having the largest effects on the development of plant seeds. Although environmental factors (drought, salt, and extreme temperature) limit seed formation, the regulation of gene expression during seed maturation is the ultimate determinant of the fate of developing seeds [5]. There is thus a need to study the mechanism and regulatory network underlying the processes of seed development.

The development of the seed requires a series of complex and dynamic changes in cell division and differentiation, as well as in the biosynthesis of hormones, carbohydrates, proteins, cell walls, lipids, amino acids, and secondary metabolites. Critical to these processes is the expression and regulation of a large number of genes. Thus, the study of the expression patterns of these genes can enhance our understanding of the molecular mechanisms underlying the accumulation of various nutrients during seed development. Previous studies have identified various genes that play key roles in seed development [6], including genes that control embryo generation and differentiation [7], endosperm formation and development [8], seed coat formation [9], and seed size [10]. Many studies have examined the synthesis and accumulation of nutrients, such as genes encoding seed storage proteins, lipid biosynthesis catabolism and storage, early starch synthesis, and late transformation [11–13].

The plant hormones ABA and GAs have important roles in seed development. ABA regulates many developmental processes in plants, including seed maturation and dormancy, nodule development, and plant senescence [14, 15]. Higher plants use the carotenoid pathway initiated by *β*-carotene to synthesize ABA [16]. GA is a diterpenoid plant hormone biosynthesized through complex pathways that controls all aspects of plant growth and development, including seed germination, stem elongation, leaf unfolding, and flower and seed development [17]. Several studies have shown that ABA and GAs (mostly GA_1_, GA_3_, GA_4_ and GA_7_ in higher plant) antagonistically regulate diverse aspects of plant growth, and the dynamic balance between these hormones is key to the regulation of seed development [18]. ABA is necessary for inducing the cessation of germ cell division and maintaining seed dormancy, and GA is necessary for ending dormancy and initiating seed germination [19].

Alfalfa (*Medicago sativa*) is a high-yield and top-quality forage with high protein content; it is thus known as ‘the queen of forage’. Alfalfa hay is the most important roughage for herbivorous livestock such as cattle and sheep [20]. In addition, alfalfa buds are a natural alkaline food that is low in calories and rich in various amino acids and vitamins; the buds are also known to promote the circulation of blood and prevent several diseases [21]. Research on the development of alfalfa seeds is important for increasing seed yield, the rapidity of seed propagation, and seed quality. Studies examining the role of ABA, GA_1_, GA_3_, GA_4_ and GA_7_ in seed development at the transcriptional level have been mostly conducted on plants other than alfalfa [22, 23]. Consequently, transcriptomic changes during the development of alfalfa seeds and the role of hormone-related gene regulation in the seed development process remain unclear. There is thus a need to clarify the regulatory mechanisms underlying alfalfa seed development. In this study, a transcriptome analysis was carried out on alfalfa seeds at 11, 19, 27, 35, and 43 days after pollination (DAP). The content of ABA, GA_1_, GA_3_, GA_4_ and GA_7_ were determined, and the hormone biosynthesis pathways were determined. Changes in gene expression were determined, and the transcriptome data were validated by quantitative real-time PCR (qRT-PCR). The results of this study enhance our understanding of the molecular mechanism underlying hormone-related gene regulation during seed development at the transcriptional level.

## Results

### Seed development stages and morphological features

**Fig. 1:**
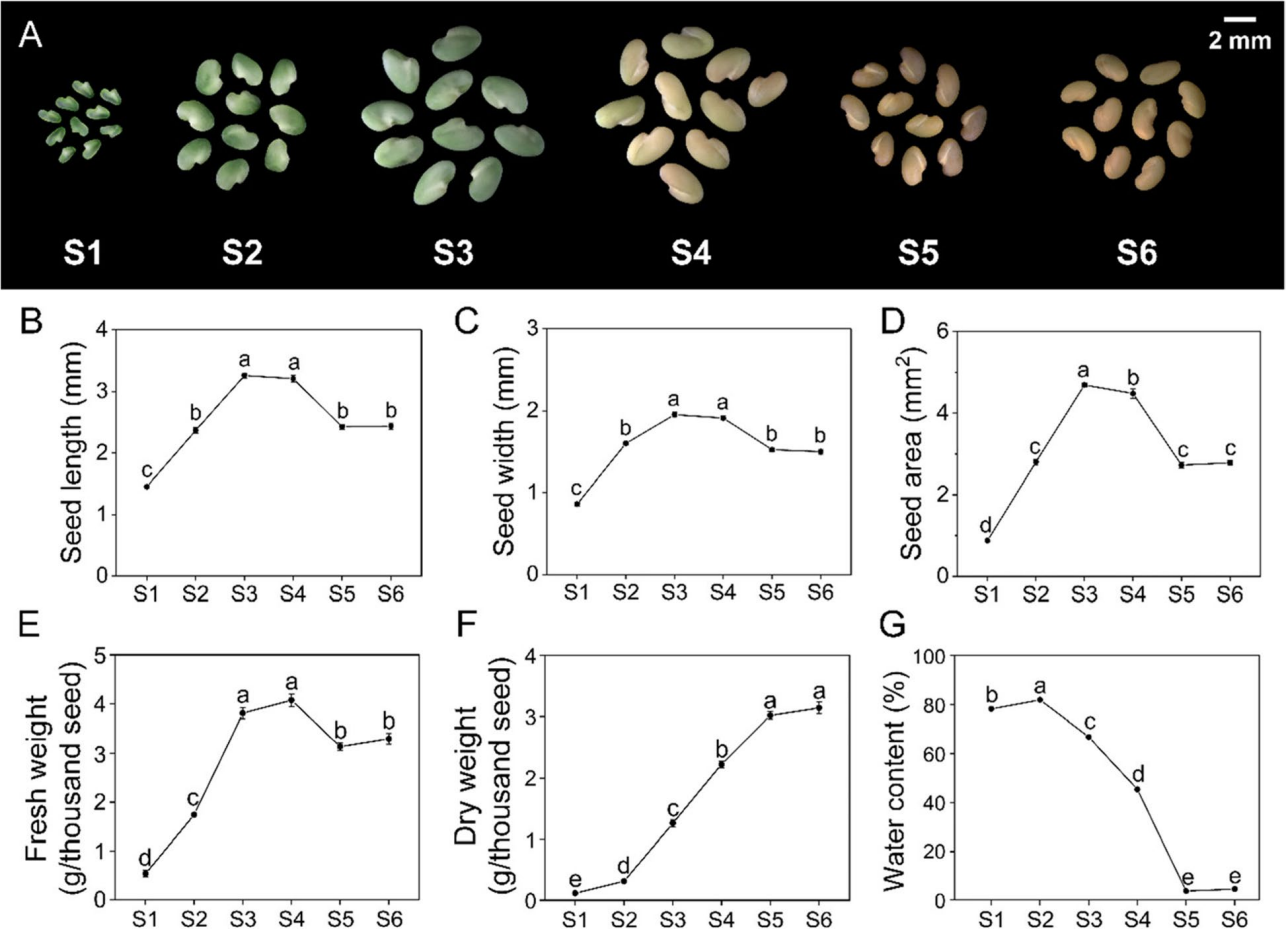
Observation and measurement of the morphology of alfalfa seeds at five stages of development. **A** The developmental progress of alfalfa seeds. Length (**B**), width (**C**), area (**D**), fresh weight (**E**), dry weight (**F**), and water content (**G**) of developing seeds. Alfalfa seeds were obtained at 11, 19, 27, 35, 43, and 51 days after pollination, and the time points were designated as S1, S2, S3, S4, S5, and S6, respectively. The scale bar is 2 mm. Different letters indicate significant differences among stages (Duncan’s multiple tests, ANOVA, *P* < 0.05)

The six stages of alfalfa seed development from pollination to full maturity are shown in Fig. 1. At stage 1 (S1, 11 DAP) and stage 2 (S2, 19 DAP), the seed coat was smooth and could be easily crushed with fingertips. At stage 3 (S3, 27 DAP), the seed coat was slightly rough, and the seeds were hard but could be easily broken with fingernails. From stage 4 (S4, 35 DAP) to stage 5 (S5, 43 DAP), the surface of the seed coat was rough, and the seeds were hard and difficult to break with fingernails (Fig. 1A). During these five stages of seed development, the seed coat changed from bright green (S1, S2) to light green (S3), gradually to yellowish-green (S4), and finally to their normal colour (S5) (Fig. 1A). The seed size first increased and reached a maximum at S3; it then gradually decreased as nutrients accumulated in the late stage of seed maturation (Fig. 1A-D). The fresh weight first increased, then decreased, and reached a maximum at S4 (Fig. 1E). The dry weight increased continuously, indicating that nutrients gradually accumulated (Fig. 1F). The water content of the seeds reached a maximum at S2 and then decreased from S2 to S6 (Fig. 1G). No significant changes in morphology and physiology were observed between stage 6 (S6, 51 DAP) and S5, which suggested that full maturity was reached at S5 (Fig. 1). RNA-Seq data were generated for each of the five different stages.

### Transcriptome sequencing and assembly

A total of 15 cDNA libraries from the five stages of seed development (S1, S2, S3, S4, and S5) were constructed for Illumina sequencing to comprehensively analyse the seed development and hormone biosynthesis and accumulation at the transcriptional level. There were three biological replicates at each stage. A total of 656,647,958 raw reads and 640,383,114 clean reads were obtained, which consisted of 96.08 Gb clean bases (Additional file 1: Table S1). The average content of GC was 42.22%. The average values of Q20 and Q30 were 98.28 and 94.50%, respectively (Additional file 1: Table S1). The clean reads in each library were mapped to the reference genome of alfalfa [24]. The proportion of clean reads mapped to the reference genome ranged from 73.66 to 80.88%, and the percentage of uniquely mapped reads was over 69.00% (Additional file 1: Table S1).

**Fig. 2:**
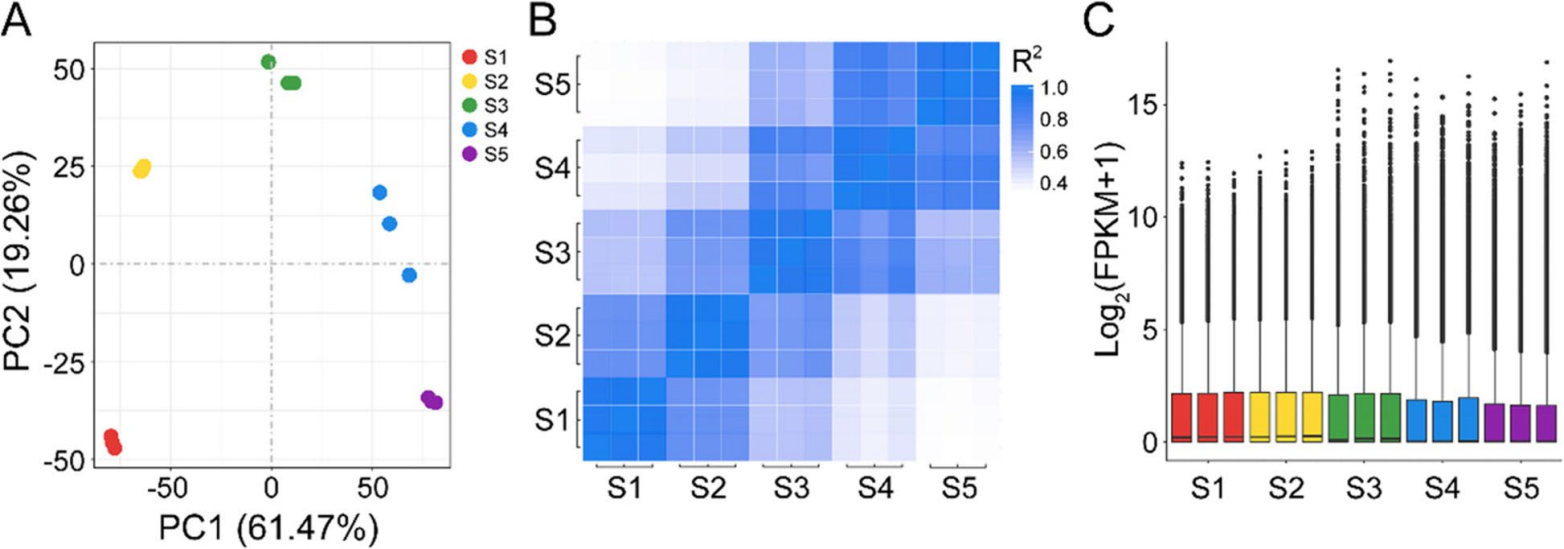
Statistical analysis of sequencing data. **A** Principal component analysis (PCA) of samples across all seed development stages revealed clear differences in gene expression levels among samples. The RNA-Seq data for each time point were based on three biological replicates. **B** Pearson’s correlation coefficients among samples. **C** Total gene expression in each sample based on the log2 transformation of FPKM values plus 1. Alfalfa seeds were obtained at 11, 19, 27, 35, and 43 days after pollination, and the time points were designated as S1, S2, S3, S4, and S5, respectively

Principal component analysis (PCA) was conducted on all samples across the different stages. The samples from different biological replicates were clustered in different groups corresponding to the distinct development stages of the seeds (Fig. 2A). Pearson’s correlation correlations were used to evaluate relationships between biological replicates. The correlations between replicates ranged from 0.93 to 0.97, and the correlations between different stages were between 0.35 and 0.88, indicating that the results were highly repeatable (Fig. 2B). The distribution of the gene expression levels of different samples is shown in Fig. 2C.

### qRT-PCR validation

**Fig. 3:**
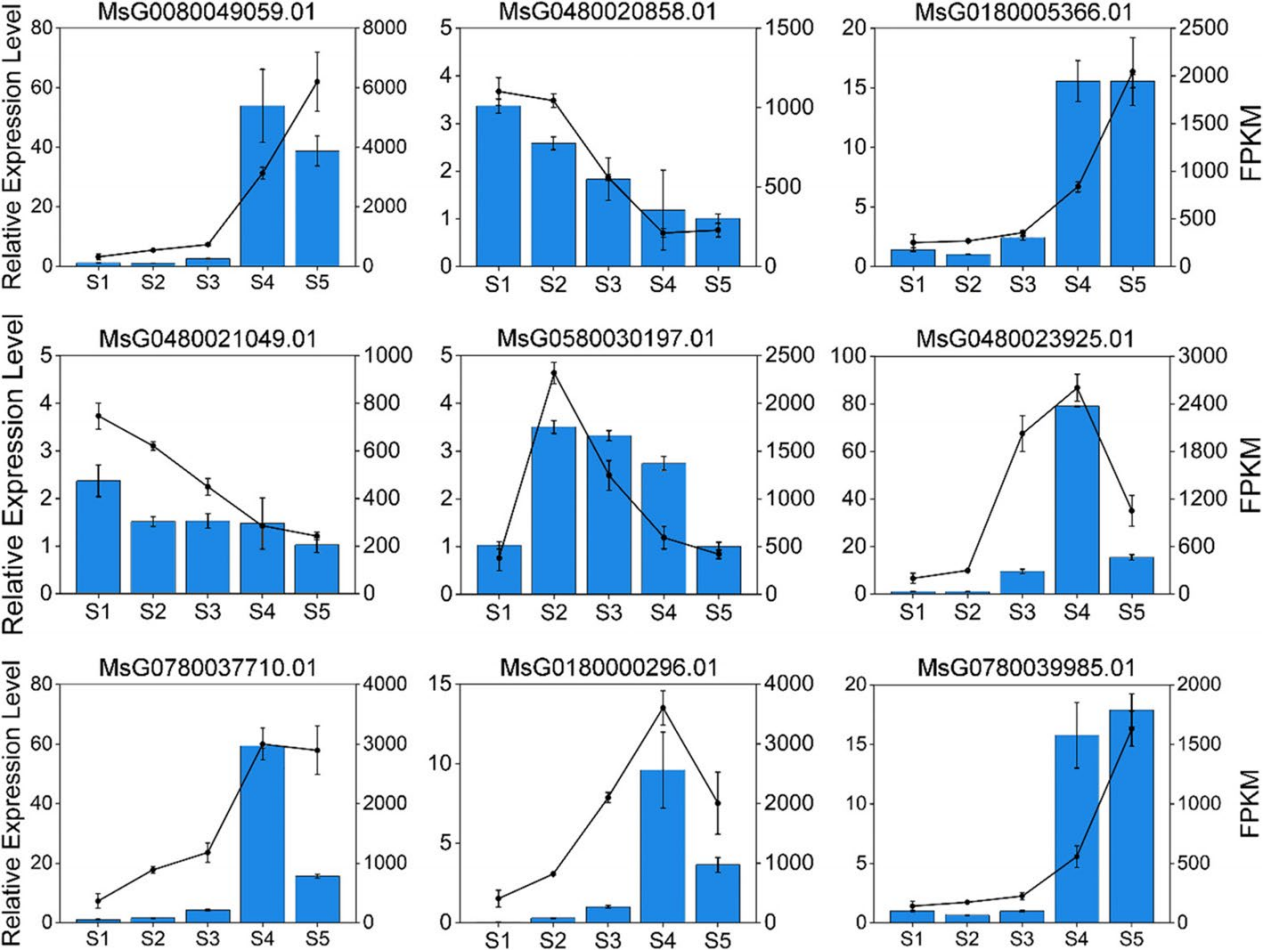
The FPKM values and relative expression levels of nine randomly selected DEGs in alfalfa seeds at five development stages. Bars indicate the level of relative expression based on the qRT-PCR results (left Y-axis). Plots show the transcript abundance change (FPKM) based on the results of the RNA-Seq analysis (right Y-axis). Alfalfa seeds were obtained at 11, 19, 27, 35, and 43 days after pollination, and the time points were designated as S1, S2, S3, S4, and S5, respectively

To validate the DEGs identified by RNA-Seq, nine DEGs were randomly selected for qRT-PCR assays. The qRT-PCR results were consistent with the RNA-Seq data, which indicated that the RNA-Seq data were reliable and accurate (Fig. 3).

### DEGs during alfalfa seed development

**Fig. 4:**
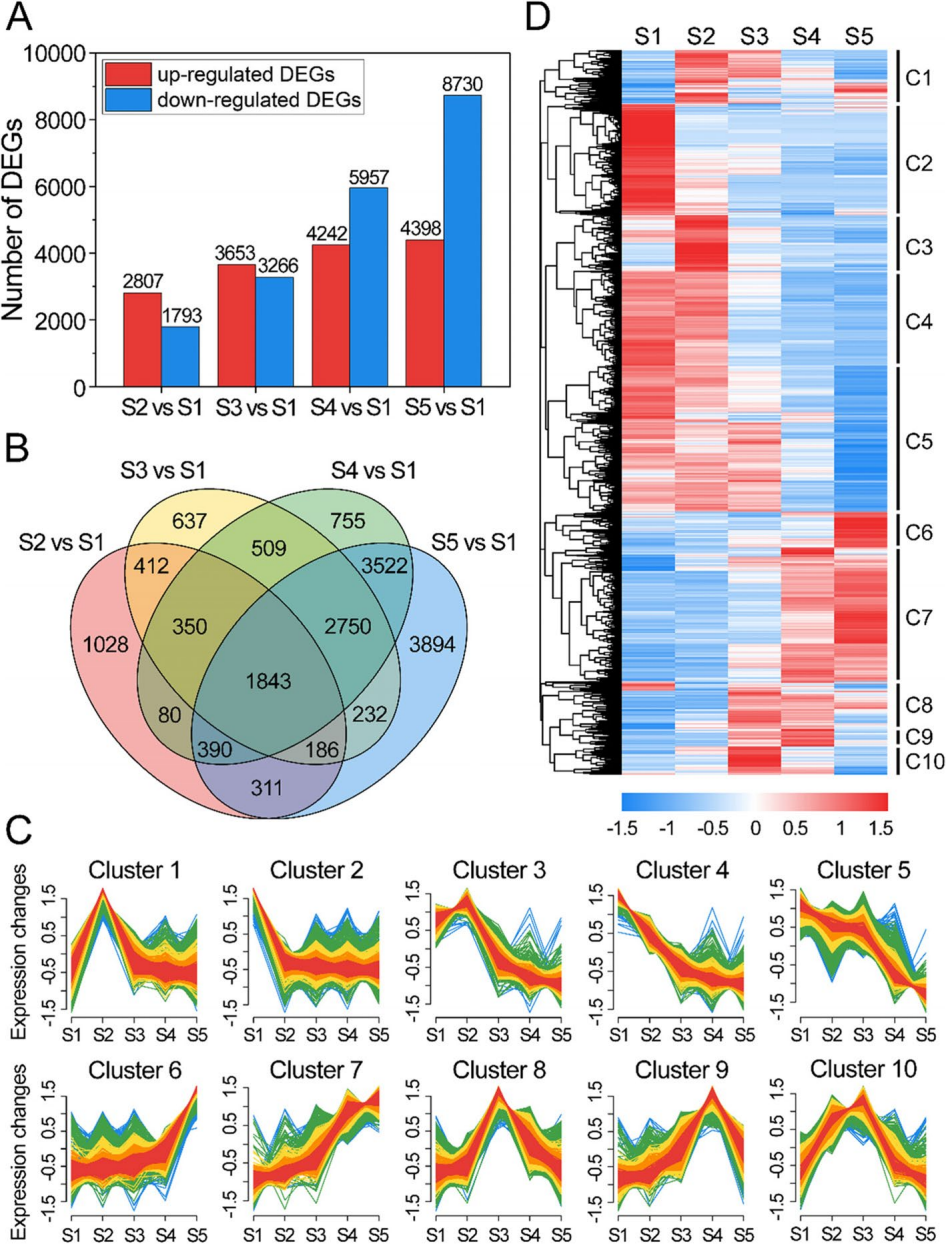
Differentially expressed genes (DEGs) during seed development. **A** The number of up- and down-regulated DEGs for each comparison group. **B** Venn diagrams showing the unique and common DEGs in various comparison groups (S2 vs. S1; S3 vs. S1; S4 vs. S1; and S5 vs. S1). **C** Cluster analysis of the expression profiles for DEGs at five stages of seed development. The Y-axis shows normalized fold-changes in gene expression, and the X-axis indicates each stage; there were three independent biological replicates for each time point. **D** Heatmap of the normalized abundances of transcripts in alfalfa seeds during each development stage. Alfalfa seeds were obtained at 11, 19, 27, 35, and 43 days after pollination, and the time points were designated as S1, S2, S3, S4, and S5, respectively

The FPKM method was used to calculate the expression levels of the transcripts. A total of 16,899 DEGs were identified across the five stages of seed development (Additional file 2: Fig. S1). A total of 4600 (2807 up-regulated and 1793 down-regulated), 6919 (3653 up-regulated and 3266 down-regulated), 10,199 (4242 up-regulated and 5957 down-regulated), and 13,128 (4398 up-regulated and 8730 down-regulated) DEGs were identified for S2 vs. S1, S3 vs. S1, S4 vs. S1, and S5 vs. S1, respectively (Fig. 4A). Of these DEGs, 1028, 637, 755, and 3894 were specific to S2, S3, S4, and S5, respectively (Fig. 4B). A total of 1843 DEGs exhibited different expression patterns in different stages, indicating that the expression of these DEGs changed continuously during the development of alfalfa seeds (Fig. 4B). Analysis of the expression patterns of all DEGs revealed 10 expression patterns with significantly different expression levels among the 16,899 DEGs (Fig. 4C), including seven up-regulation patterns (clusters 1, 2, 3, 4, 6, 8, and 9) and three down-regulation patterns (clusters 5, 7, and 10). The expression levels of clusters 4 and 8 peaked at S2, the expression levels of clusters 3 and 6 peaked at S3, and the expression levels of cluster 2 peaked at S4. Hierarchical clustering results of all 16,899 DEGs were consistent with the clustering results of the expression patterns (Fig. 4D).

### GO and KEGG pathway enrichment analysis of the DEGs

**Fig. 5:**
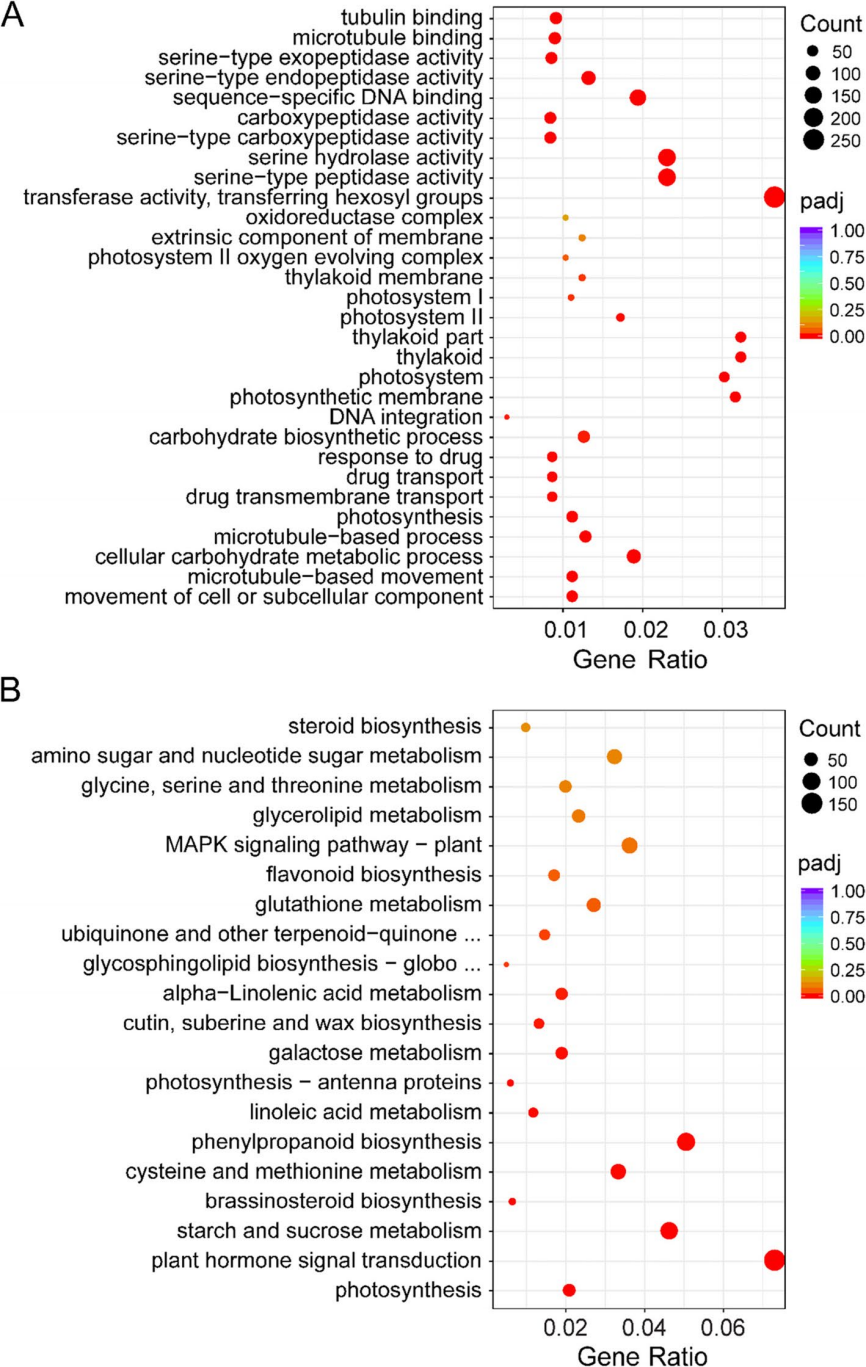
Analysis of GO terms and KEGG pathways of differentially expressed genes (DEGs) in alfalfa seeds at five development stages. **A** GO enrichment analysis. **B** KEGG enrichment analysis. The left Y-axis indicates the GO term and KEGG pathway. The X-axis indicates the gene ratio. High padj values are indicated in purple, and low padj values are indicated in red

Gene Ontology (GO) enrichment analysis was performed to classify the functions of DEGs. In total, 102 GO terms were functionally classified into three main categories: biological processes (36 members), cellular components (7 members), and molecular functions (59 members). In the biological processes category, movement of cell or subcellular component (GO:0006928, padj = 0.00012), microtubule-based movement (GO:0007018, padj = 0.00012), and cellular carbohydrate metabolic process (GO:0044262, padj = 0.00017) were significantly enriched. In the cellular components category, processes related to photosynthesis were significantly enriched, including photosynthetic membrane (GO:0034357, padj = 0.00001), photosystem (GO:0009521, padj = 0.00001), and thylakoid (GO:0009579, padj = 0.00001). In the molecular function category, processes related to enzyme activity were significantly enriched, such as transferase activity, transferring hexosyl groups (GO:0016758, padj< 0.00001), serine-type peptidase activity (GO:0008236, padj< 0.00001), and serine hydrolase activity (GO:0017171, padj< 0.00001) (Fig. 5A).

An enrichment analysis was performed using the Kyoto Encyclopedia of Genes and Genomes (KEGG) database [25] to identify the catabolism pathways in which the DEGs were involved. Of all 123 pathways, the most representative pathways included ‘photosynthesis’ (mtr00195, 56 genes, padj< 0.00001), ‘plant hormone signal transduction’ (mtr04075, 284 genes, padj = 0.00001), and ‘starch and sucrose catabolism’ (mtr00500, 173 genes, padj = 0.00019) (Fig. 5B and Additional file 1: Table S2). ‘Cutin, suberine and wax biosynthesis’ (mtr00073, padj = 0.00480) was significantly enriched in S2 vs S1, indicating that it plays an important role in the formation of seed structure (Additional file 2: Fig. S2). Several pathways involved in nutrient mobilization were enriched in S4 vs S1 and S5 vs S1, such as ‘starch and sucrose metabolism’, ‘Linoleic acid metabolism’ (mtr00591, padj = 0.00134) and ‘galactose metabolism’ (mtr00052, padj = 0.00216). It shows that the synthesis and accumulation of nutrients begin in the later stage of seed development (Additional file 2: Fig. S2). ‘Plant hormone signal transduction’ was significantly enriched in all four comparisons, indicating that plant hormones have sustained and significant regulatory effects during alfalfa seed development (Additional file 2: Fig. S2).

### DEGs involved in ABA and GA biosynthesis and catabolism

**Fig. 6:**
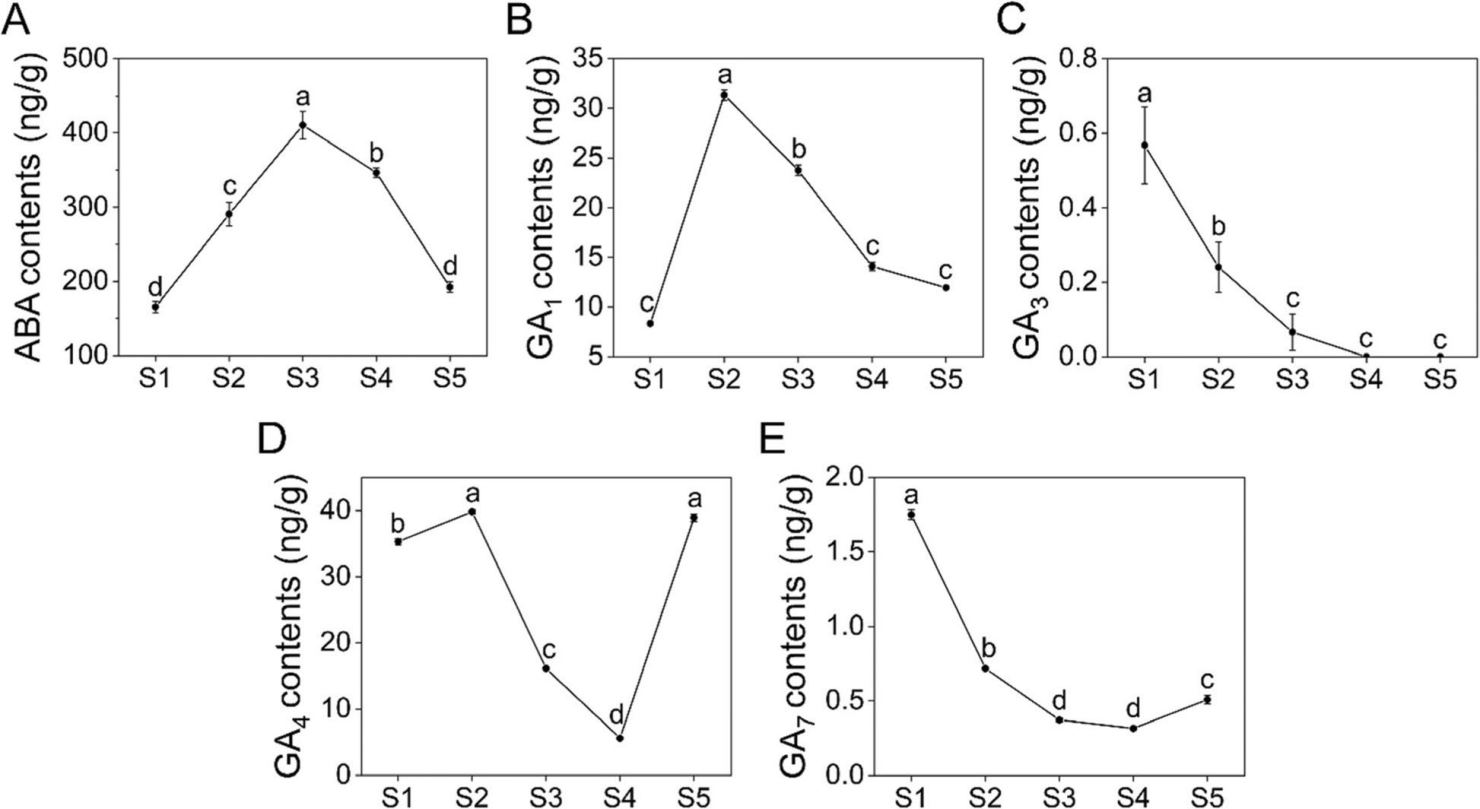
Changes in plant hormone content during alfalfa seed development. **A** ABA, **B** GA_1_, **C** GA_3_, **D** GA_4_ and **E** GA_7_ content at different stages. All data shown indicate the average mean of three biological replicates (*n* = 3). The different letters indicate significant differences among stages (Duncan’s multiple tests, ANOVA, *P* < 0.05). Alfalfa seeds were obtained at 11, 19, 27, 35, and 43 days after pollination, and the time points were designated as S1, S2, S3, S4, and S5, respectively

To investigate the roles of endogenous hormones in seed development, the content of ABA, GA_1_, GA_3_, GA_4_ and GA_7_ was measured at different stages (Fig. 6). Both ABA and GA_1_ levels increased first and then decreased. The ABA level reached a peak at S3, while the GA_1_ level reached a peak at S2 (Fig. 6A-B). The content of GA_3_ decreased continuously during seed maturation and was nearly zero in S4 and S5 (Fig. 6C). In contrast to GA_1_ and GA_3_, GA_4_ and GA_7_ levels increased significantly at late stage of seed development (S5), and GA_4_ even peaked at S5 (Fig. 6D-E).

**Fig. 7:**
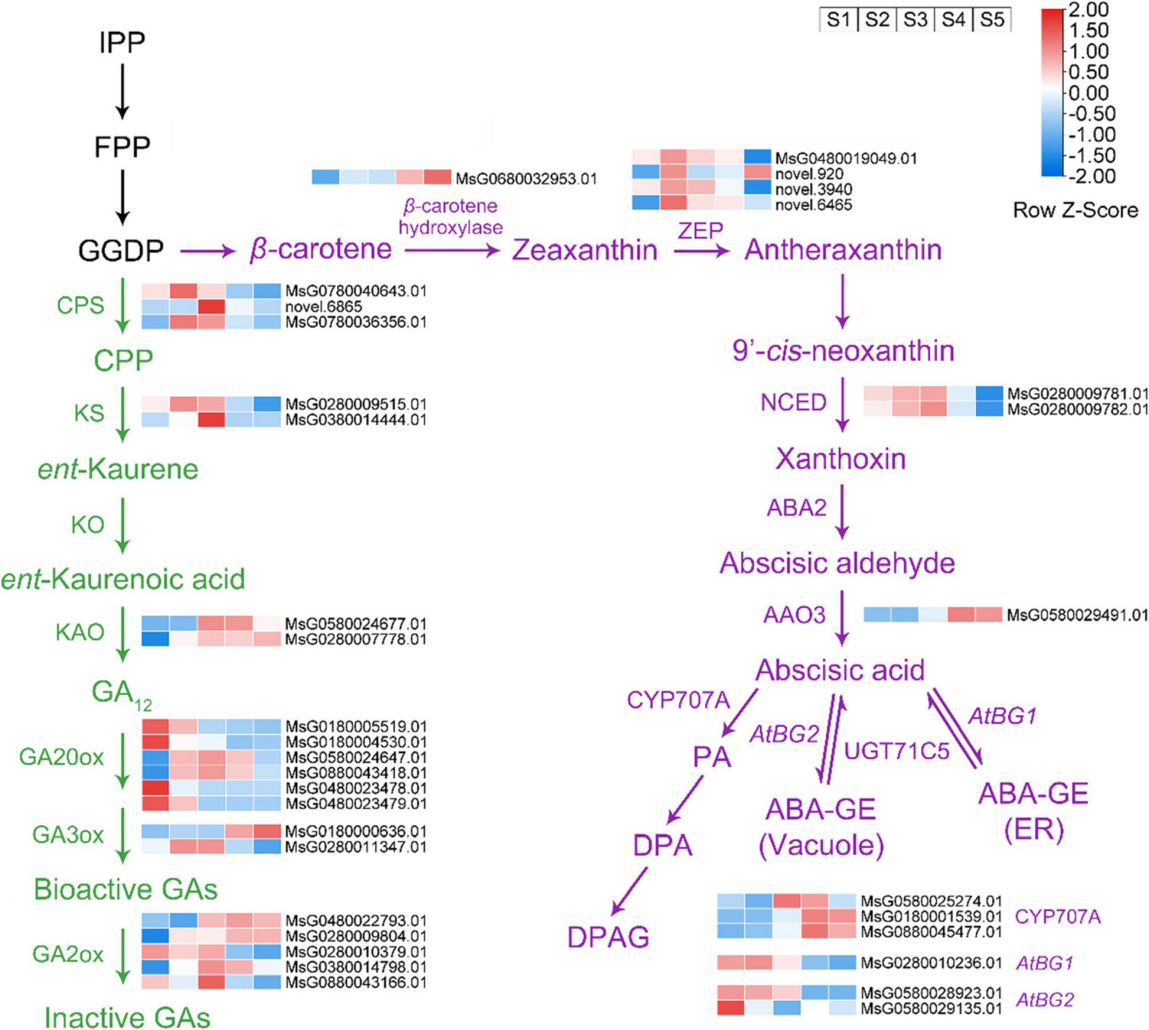
DEGs associated with ABA (purple words) and GAs (green words) during alfalfa seed development stages. Changes in expression levels are indicated by changes in colour; blue indicates lower expression levels, whereas red indicates higher expression levels. The expression levels of relevant genes were log2 transformed. The colour of each block is based on the expression level of genes and the time points were designated as S1, S2, S3, S4 and S5. The sample names are indicated at the top of the figure. Abbreviations: IPP: isopentenyl diphosphate. FPP: farnesyl pyrophosphate. GGDP: geranylgeranyl diphosphate. ZEP: zeaxanthin epoxidase. NCED: 9′-*cis*-epoxycarotenoid dioxygenase. ABA2: ABA deficient 2. AAO3: abscisic aldehyde oxidase 3. PA: phaseics acid. DPA: dihydrophaseic acid. DPAG: dihydrophaseic acid-4-O-β-D-glucoside. *AtBG*: *Arabidopsis thaliana* beta-1,3-glucosidase. ABA-GE: ABA-glucose ester. CPS: *ent*-copalyl diphosphate synthase. CPP: copalyl diphosphate. KS: *ent*-kaurene synthase. KO: *ent*-kaurene oxidase. KAO: *ent*-kaurenoic acid oxidase. GA2/3/20ox: gibberellin 2/3/20 oxidase

We next analysed the genes proposed to be involved in the ABA and GA pathways (Fig. 7). A total of 14 DEGs related to ABA biosynthesis and catabolism pathways were identified from the library, and their expression patterns were analysed (Additional file 1: Table S3). A gene encoding *β*-carotene hydroxylase was highly expressed and peaked in late development stages (S5). The expression of four genes encoding zeaxanthin epoxidase (ZEP) was up-regulated, peaked at S2, and then decreased. Two genes encoding 9′-*cis*-epoxycarotenoid dioxygenases (NCED) were highly expressed during seed development (S2 and S3). The expression of one gene encoding abscisic aldehyde oxidase 3 (AAO3) peaked at S4 and S5. Three *CYP707A*s were found to be involved in ABA catabolism pathways, and the expression of these genes was up-regulated in later stages of seed development (S4 or S5). One *AtBG1* and two *AtBG2* genes were identified, and the expression of these genes peaked in the early stages of seed development (S1 or S2).

The expression of 20 DEGs related to GA biosynthesis and catabolism pathways was analysed (Additional file 1: Table S3). The expression of genes encoding *ent-*copalyl diphosphate synthase (CPS), *ent-*kaurene synthase (KS), and *ent*-kaurenoic acid oxidase (KAO) was up-regulated Gibberellin 3/20 oxidase (GA3/20ox) is important for the oxidation of GA_12_ to other types of GAs, and GA20ox is the key enzyme in GA biosynthesis. A total of six genes encoding GA20ox were identified, and the expression of four of them (*MsG0180005519.01*, *MsG0180004530.01*, *MsG0480023478.01*, and *MsG0480023479.01*) was down-regulated and peaked in the earliest stage of seed development (S1). The expression levels of two genes (*MsG0580024647.01* and *MsG0880043418.01*) first increased and then decreased; the highest expression level was observed at S3. Two genes encoding GA3ox were identified (*MsG0180000636.01* and *MsG0280011347.01*), and the expression of these genes were up-regulated during seed development. Five gibberellin 2 oxidase (GA2ox) candidate genes were identified, and these genes play a role in GA catabolism.

## Discussion

### Morphological and physiological changes in seed development

The seed development of legumes has been examined in several studies. In soybean, the seed coat changes from bright green to light green and finally becomes yellow; the fresh weight first increases, then decreases, and peaks in the middle stage of seed development [23]. During the seed development of *Medicago truncatula*, the seed dry weight increases continuously and decreases slightly in the final stage of seed development [22]. Our findings are consistent with these studies and indicate that dramatic physiological changes occur during alfalfa seed development; additional studies of these changes at the molecular level are needed.

### Biosynthesis and catabolism of ABA in alfalfa seeds

The physiological effects of ABA are regulated by its endogenous levels [26]. During the seed development of Arabidopsis and *Nicotiana plumbaginifolia*, the level of ABA increases during the early stage, peaks in the middle stage of seed development, and then decreases during seed desiccation [27, 28]. These findings are consistent with the results of our research.

Plants synthesize ABA through the carotenoid pathway. In the process of *β*-carotene turning into zeaxanthin, the *β*-carotene hydroxylase acts a very important function [29]. Although the content of ABA is low in the early stage of alfalfa seed development, its precursors are synthesized during these stages, and *β*-carotene is one of the important precursors of ABA biosynthesis. The *β*-carotene hydroxylase candidate gene (*MsG0680032953.01*) was highest in the later stage of seed development. One possible explanation for this pattern is that alfalfa seeds need to accumulate ABA in the later stage of development.

Conversion of zeaxanthin to antheraxanthin is catalyzed by ZEP, which is the first rate-limiting step of ABA biosynthesis [30]. Four genes encoding ZEP were highly expressed in the early stage of seed development (S2), indicating that the rate of cleavage of zeaxanthin was high, which contributed to the accumulation of xanthoxins in the early stage of seed development and generated the precursors necessary for the biosynthesis and accumulation of ABA in the middle and late stages of seed development [31].

The 9′-*cis*-neoxanthin synthesized in the previous step can be oxidized by NCED to produce xanthoxins [32], which is the second rate-limiting step of ABA biosynthesis [33]. The NCED gene family is thought to play a direct role in determining the final ABA content of seeds and the response induced by ABA [26]. The expression of NCED candidate genes (*MsG0280009781.01* and *MsG0280009782.01*) was higher in the middle stage of seed development, which was consistent with the changes in the ABA content, indicating that NCED plays a key role in regulating ABA biosynthesis [26].

In the cytoplasm, short-chain alcohol dehydrogenase encoded by *ABA-DEFICIENT 2* (*ABA2*) converts xanthoxin to abscisic aldehyde, which is finally oxidized to abscisic acid by AAO3 [34]. The AAO3 candidate gene (*MsG0580029491.01*) was up-regulated, and its expression was higher in the later stage of seed development, which is consistent with the results of previous studies [35].

There are two main types of ABA catabolism. One is the decomposition of ABA into DPA-4-O-β-D-glucoside (DPAG), in which cytochrome P450 monooxygenase encoded by *CYP707A* acts as a catalyst [36]; the other is the glycosylation of ABA by UDP-glucosyltransferase encoded by *UGT71C5* to form ABA-glucose ester (ABA-GE), which is the inactive form of ABA. *β*-glucosidases encoded by *AtBG1/2* can convert ABA-GE to active ABA when needed by the plant [37]. The three *CYP707A* candidate genes identified in this study were highly expressed in the middle and later stages of seed development, and one *AtBG1* and two *AtBG2* candidate genes were highly expressed in the early stage of seed development. ABA might maintain a suitable internal environment for seed development in the early stage of seed development through a flexible transformation mechanism involving a cycle of *β*-glucosidases encoded by *AtBG1/2* and UDP-glucosyltransferase encoded by *UGT71C5*. However, in the later stage of seed development, the seeds mainly depend on *CYP707A* to completely decompose the redundant ABA, which makes the seeds mature and dormant [38].

### Biosynthesis and catabolism of GAs in alfalfa seeds

GA_1_, GA_3_, GA_4_ and GA_7_ are considered to be the most important GA in higher plants, and seeds are major sites of GA synthesis [39]. At the early stage of alfalfa seed development (S1-S2), the contents of four GAs were all at a high level, which was consistent with the results of Arabidopsis [40]. Therefore, it can be considered that S1-S2 is the key period for GAs synthesis of alfalfa seeds. At the middle stage (S2-S4), the contents of the four GAs decreased continuously, indicating that S2-S4 may be the key stage of endogenous GA metabolism in alfalfa seeds. At the late stage of seed development (S5), the contents of GA_1_ and GA_3_ decreased, indicating that the requirement of these two hormones was greatly reduced when alfalfa seeds entered the stage of maturation and dehydration [41]. On the contrary, amount of GA_4_ and GA_7_ accumulated in seeds, indicates that a certain level of GA may need to be maintained at the late stage of seed development [42].

Plants synthesize GA from GGDP, which is a common precursor for ABA. GGDP is cyclized to *ent-*kaurene under the catalysis of CPS and KS. Three CPS candidate genes (*MsG0780040643.01*, *novel.6865*, and *MsG0780036356.01*) and two KS candidate genes (*MsG0280009515.01* and *MsG0380014444.01*) were highly expressed in the early and middle stages of seed development, which resulted in the accumulation of sufficient precursors for GA_12_ biosynthesis. This result was consistent with the observed changes in the level of four GAs. *Ent-*kaurene is converted to GA_12_ through a series of reactions catalysed by *ent*-kaurene oxidase (KO) and KAO, and GA_12_ is further oxidized by GA3/20ox to generate bioactive GA [39]. Two candidate genes of KAO have been identified in the transcriptome, all of which belong to *KAO2*, indicating that KAO is a key enzyme that catalyses the conversion of *ent-*kaurenoic acid to GA_12_, which is the common precursor of bioactive GAs (including GA_1_, GA_3_, GA_4_ and GA_7_) [39, 43]. Numerous genes encoding GA20ox are downregulated during alfalfa seed development, suggesting that the synthesis of bioactive GA was reduced, which was consistent with the observation of the declined GA content .

Finally, GA2ox converts bioactive GA to inactive GA [44]. Most of the GA2ox candidate genes were highly expressed in the middle and later development stages, indicating that amount of inactive GA was accumulated in the later stage of alfalfa seed development, which was consistent with GA_1_, GA_3_ and GA_7_ levels [41].

### Dynamic regulation of ABA and GA during alfalfa seed development

ABA and GA are recognized as primary hormones that antagonistically regulate seed development. A large number of genes were upregulated during alfalfa seed development in GA biosynthesis pathway, which was not concomitant with the declination of GA content. One possible reason is that these genes were also related to ABA synthesis and contributed to ABA accumulation at the early stage of seed development. For example, isopentenyl diphosphate (IPP), farnesyl pyrophosphate (FPP), and geranylgeranyl diphosphate (GGDP) are the common precursors in the biosynthesis pathways of ABA and GA, which may provide more allocation for ABA synthesis, which also led to less allocation for GA synthesis. Therefore, although genes related to GA synthesis were up-regulated, the content of GA at the physiological level dropped in the early seed development.

## Conclusions

The morphological and physiological characteristics of alfalfa seeds, the ABA and GA_3_ content, and the gene expression profiles were analysed during the development of seeds to provide information that enhances our understanding of hormone regulation in alfalfa seed development. The morphology of alfalfa seeds, seed dry and fresh weight, and seed water content were determined at the five stages of seed development (11, 19, 27, 35, and 43 DAP). Illumina sequencing technology was used for the transcriptome analysis, and a total of 16,899 DEGs were identified. All DEGs were divided into 10 clusters according to their expression patterns. The GO terms and KEGG pathways significantly enriched for these DEGs were also determined. In addition, 14 genes involved in ABA biosynthesis and 20 genes involved in GA_3_ biosynthesis were identified, and these might be key regulators in different stages of alfalfa seed development. To the best of our knowledge, this is the first study to characterize the transcriptional changes in alfalfa seeds during their development. The findings of this study enhance our understanding of the complex transcriptome dynamics and gene regulation mechanisms during the process of seed development and hormone regulation. Our results also have implications for improving the seed yield of alfalfa.

## Methods

### Plant material

Seeds of alfalfa (variety Gold Empress) were collected from a germplasm resources nursery in Yuzhong, Lanzhou, China. After pollination, seeds were hand-collected at intervals of 8 days (11, 19, 27, 35 43, and 51 DAP) from the beginning of pollination until full maturity from June to August 2020. There were six biological replicates for each stage (three for transcriptome sequencing and three for hormone content determination) with 1 g of seeds per replicate. All the biological replicates were collected from the same three individuals. The collected samples were flash-frozen in liquid nitrogen and stored in a − 80 °C refrigerator.

### Morphological and physiological determination

To reduce random error, the following analyses were all based on 200 seeds collected from each of the three individuals at each stage. After the pods were removed on ice, seeds were immediately photographed with a stereomicroscope (Zeiss SteREO Discovery V20, Germany), and the length, width, and projected area were measured by Digimizer Image Analysis Software (https://www.digimizer.com/). The fresh weight of seeds was measured immediately after the pods were removed, and then the seeds were dried at 85 °C until a constant weight was achieved. Each measurement was repeated three times, and the water content was calculated by subtracting the dry weight from the fresh weight.

### Library construction and sequencing

One microgram of RNA was used to prepare samples. mRNA was purified from total RNA using poly-T oligo-attached magnetic beads. First-strand cDNA was synthesized using random hexamer primers and M-MuLV Reverse Transcriptase (RNase H). Second-strand cDNA synthesis was performed using DNA Polymerase I and RNase H. Remaining overhangs were converted into blunt ends via exonuclease/polymerase activities. The library fragments were purified with the AMPure XP system (Beckman Coulter, Beverly, USA). PCR was then performed using Phusion High-Fidelity DNA polymerase, Universal PCR primers, and Index (X) Primer. PCR products were purified (AMPure XP system), and library quality was assessed on the Agilent Bioanalyzer 2100 system. The clustering of the index-coded samples was performed on a cBot Cluster Generation System using the TruSeq PE Cluster Kit v3-cBot-HS (Illumia) according to the manufacturer’s instructions. After cluster generation, the library preparations were sequenced on an Illumina Novaseq platform, and 150-bp paired-end reads were generated. Clean reads were obtained by removing reads containing adapter, reads containing ploy-N and low-quality reads from raw reads in fastaq format. All the downstream analyses were based on clean data with high quality. Reference genomes were indexed using Hisat2 v2.0.5 and paired-end clean reads were aligned to the reference genome. FeatureCounts v1.5.0-p3 was used to count the reads numbers mapped to each gene. And then FPKM of each gene was calculated based on the length of the gene and reads count mapped to this gene.

### Differentially expressed genes (DEGs) analysis

Differential expression analysis of five stages (three biological replicates per stage) was performed using the DESeq2 R package (1.16.1). Both the absolute values of the log_2_(Fold Change) ≥2 and the absolute values of the padj ≤0.05 were used as the thresholds for identifying significant DEGs. Cluster analysis and expression profile evaluation were carried out using the hierarchical clustering and K-means clustering methods in MEV4.9 software (https://sourceforge.net/projects/mev-tm4/files/mev-tm4/) [45]. GO enrichment analysis of DEGs was conducted using the clusterProfiler R package, and gene length bias was corrected. GO terms were considered significantly enriched for DEGs at *P* < 0.05. KEGG pathway enrichment analysis of DEGs was conducted using KOBAS 3.0 (http://kobas.cbi.pku.edu.cn/) [46].

The location and annotation information of clean reads were obtained by comparing the RNA-Seq data and reference genome of ZhongMu No.1. Combined with the published hormone-related gene information, 14 ABA-related DEGs and 20 GA-related DEGs were identified.

### Hormone content determination

The methods for quantitatively analysing the content of phytohormones were based on those described in a previous study [47]. Approximately 0.5 g of seeds were ground and mixed with the extraction buffer composed of isopropanol, ultrapure water and concentrated hydrochloric acid (2:1:0.002 by volume). The extract was shaken at 4 °C for 30 min. Then, 10 mL of dichloromethane was added, and the sample was again shaken at 4 °C for 30 min. The sample was centrifuged at 13,000 rpm for 5 min at 4 °C and then the organic phase was extracted. The organic phase was dried under N2, dissolved in methanol (0.1% methanoic acid) and filtered through a 0.22 μm filter membrane. The standard solutions with gradients of 0.1 ng/mL, 0.2 ng/mL, 0.5 ng/mL, 2 ng/mL, 5 ng/mL, 20 ng/mL, 50 ng/mL and 200 ng/mL were prepared with methanol (0.1% methanoic acid) as solvent. The purified product was subjected to high-performance liquid chromatography-tandem mass spectrometry (HPLC-MS/MS) analysis. The mobile phase A solvents consisted of methanol/0.1% methanoic acid, and the mobile phase B solvents consisted of ultrapure water/0.1% methanoic acid. The elution gradient was 0–1 min, A = 20%; 1–9 min, A = 80%; 9–10 min, A = 80%; 10–10.1 min, A = 20%; 10.1–15 min, A = 20%. The injection volume was 2 μL. MS conditions were as follows: the ionspray voltage was 4500 V; the pressure of the curtain gas, nebulizer, and aux gas were 15, 65, and 70 psi, respectively; and the atomizing temperature was 400 °C. The content of hormones was determined by an Agilent 1290 Infinity system combined with an AB Sciex QTRAP 6500 mass spectrometer. The reaction monitoring conditions were parent ions: 263.1 m/z for ABA, 347.4 m/z for GA_1_, 345.2 m/z for GA_3_, 331.4 m/z for GA_4_, 329.2 m/z for GA_7_; quantitative ions: 153.0 m/z for ABA, 259.2 m/z for GA_1_, 143.0 m/z for GA_3_, 213.1 m/z for GA_4_, 223.2 m/z for GA_7_; qualitative ions: 204.2 m/z for ABA, 273.1 m/z for GA_1_, 239.2 m/z for GA_3_, 243.2 m/z for GA_4_, 241.1 m/z for GA_7_. Measurements of each sample were taken three times.

### qRT-PCR analysis

Primer 6 software was used to design primers for qRT-PCR, and then the primers were synthesized by Tsingke Biotechnology (Beijing, China). Gene-specific primers are listed in Additional file 1: Table S4. cDNA was reverse-transcribed from total RNA using a FastQuant RT Kit (with gDNase) (Tiangen Biotech, Beijing, China). qRT-PCR analysis was performed using 2xSG Fast qPCR Master Mix (Sangon Biotech, Shanghai, China) on a CFX96 TOUCH Real-Time Detection System (Bio-Rad, Singapore) under the following parameters: 95 °C for 30 s and 40 cycles of 95 °C for 5 s and 60 °C for 30 s; each qRT-PCR experiment was conducted three times [48]. An internal control, the alfalfa *actin* gene, was used to normalize the expression data [49]. The 2^−ΔΔCt^ method was used to calculate the relative expression level of genes [50].

### Statistical analysis of data

SPSS 26.0 software was used to perform one-way analysis of variance and Duncan’s multiple range tests (*P* < 0.05). All data points were the averages of three biological replicates.

## Supplementary Information

**Additional file 1: Table S1.** Summary statistics of RNA-Seq results in seed development of *Medicago sativa.* **Table S2.** DEGs of the most representative KEGG pathways. **Table S3.** List of DEGs involved in biosynthetic and metabolism pathway of ABA and GAs. **Table S4.** qRT-PCR specific primers.

**Additional file 2: Fig. S1.** Identification of the DEGs in five development stages: S2 vs. S1 (A); S3 vs. S1 (B). S4 vs. S1 (C) and S5 vs. S1 (D). **Fig. S2.** Kyoto Encyclopedia of Genes and Genomes (KEGG) pathways in: S2 vs. S1 (A); S3 vs. S1 (B). S4 vs. S1 (C) and S5 vs. S1 (D). The left Y-axis indicates the KEGG pathway. The X-axis indicates the gene ratio. High padj values are indicated in purple, and low padj values are indicated in red.

## Data Availability

The reference genome data of Zhongmu No.1 cultivated alfalfa was obtained from https://figshare.com/articles/dataset/Medicago_sativa_genome_and_annotation_files/12623960. All transcriptome sequencing reads are available in NCBI SRA (accession number PRJNA781768).

## Abbreviations

AAO3: Abscisic aldehyde oxidase 3
ABA: Abscisic acid
ABA2: ABA-DEFICIENT 2
ABA-GE: ABA-glucose ester
*AtBG*: *Arabidopsis thaliana* beta-1,3-glucosidase
CPP: Copalyl diphosphate
CPS: *Ent*-copalyl diphosphate synthase
DAP: Days after pollination
DEG: Differentially expressed gene
DPA: dihydrophaseic acid
DPAG: dihydrophaseic acid-4-O-β-D-glucoside
FPP: Farnesyl pyrophosphate
GA: Gibberellin
GA2/3/20ox: Gibberellin 2/3/20 oxidase
GGDP: Geranylgeranyl diphosphate
GO: Gene ontology
IPP: Isopentenyl diphosphate
KAO: *Ent*-kaurenoic acid oxidase
KEGG: Kyoto encyclopedia of genes and genomes
KO: *Ent*-kaurene oxidase
KS: *Ent*-kaurene synthase
NCBI: National center for biotechnology information
NCED: 9′-cis-epoxycarotenoid dioxygenase
PA: phaseics acid
PCA: Principal component analysis
qRT-PCR: Quantitative real-time PCR
ZEP: Zeaxanthin epoxidase
