# Supplementary material for: Transcriptome analysis and identification of abscisic acid and gibberellin-related genes during seed development of alfalfa (Medicago sativa L.)
Source: BMC Genomics. 2022 Sep 13;23:651. doi: 10.1186/s12864-022-08875-0 (PMC9472388; doi:10.1186/s12864-022-08875-0)
Supplement: Supplementary file 2 — Additional file 2: Fig. S1. Identification of the DEGs in five development stages: S2 vs. S1 (A); S3 vs. S1 (B). S4 vs. S1 (C) and S5 vs. S1 (D). Fig. S2. Kyoto Encyclopedia of Genes and Genomes (KEGG) pathways in: S2 vs. S1 (A); S3 vs. S1 (B). S4 vs. S1 (C) and S5 vs. S1 (D). The left Y-axis indicates the KEGG pathway. The X-axis indicates the gene ratio. High padj values are indicated in purple, and low padj values are indicated in red. [file 12864_2022_8875_MOESM2_ESM.docx]

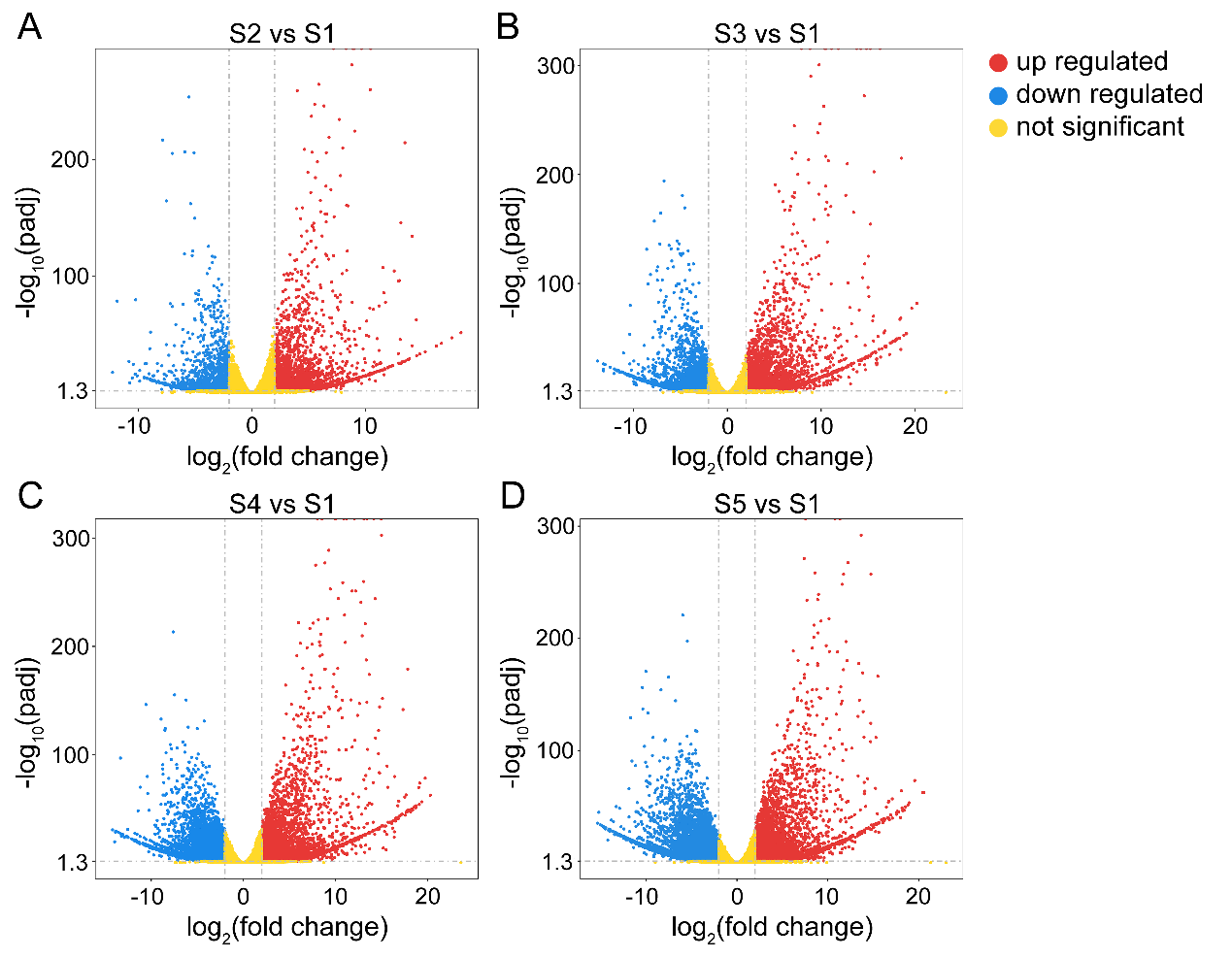


**Figure S1.** Identification of the DEGs in five development stages: S2 vs. S1 (A); S3 vs. S1 (B). S4 vs. S1 (C) and S5 vs. S1 (D).


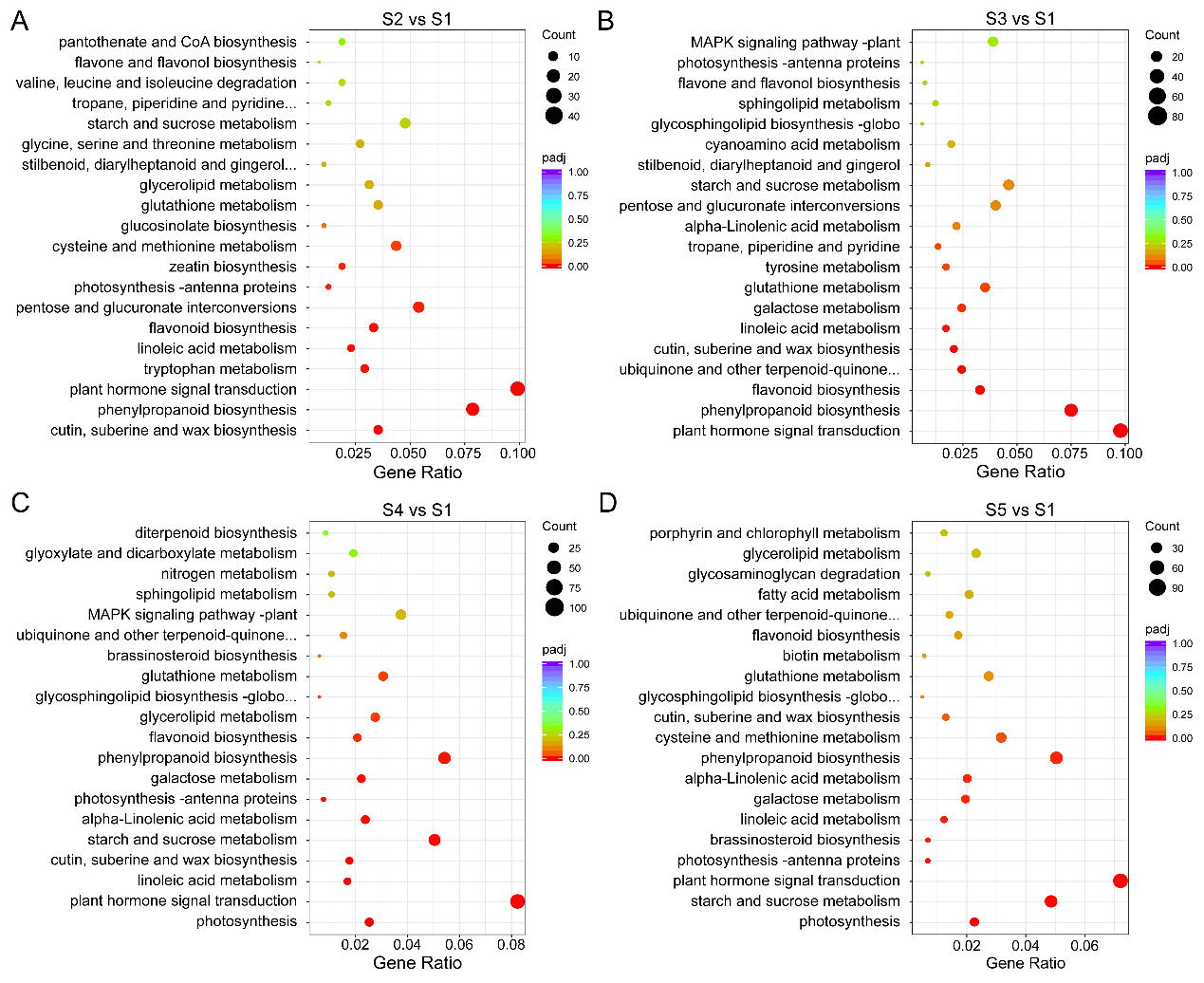


**Figure S2.** Kyoto Encyclopedia of Genes and Genomes (KEGG) pathways in: S2 vs. S1 (A); S3 vs. S1 (B). S4 vs. S1 (C) and S5 vs. S1 (D). The left Y-axis indicates the KEGG pathway. The X-axis indicates the gene ratio. High padj values are indicated in purple, and low padj values are indicated in red.
